# Supplementary material for: A male steroid controls female sexual behaviour in the malaria mosquito
Source: Nature. 2022 Jul 6;608(7921):93–7. doi: 10.1038/s41586-022-04908-6 (PMC9352575; doi:10.1038/s41586-022-04908-6)
Supplement: Supplementary file 1 — Uncropped images used in generating Fig. 2d. [file 41586_2022_4908_MOESM1_ESM.pdf]

nature portfolio

https://doi.org/10.1038/s41586-022-04908-6

Supplementary information

A male steroid controls female sexual behaviour in the malaria mosquito

In the format provided by the authors and unedited

Original scan:

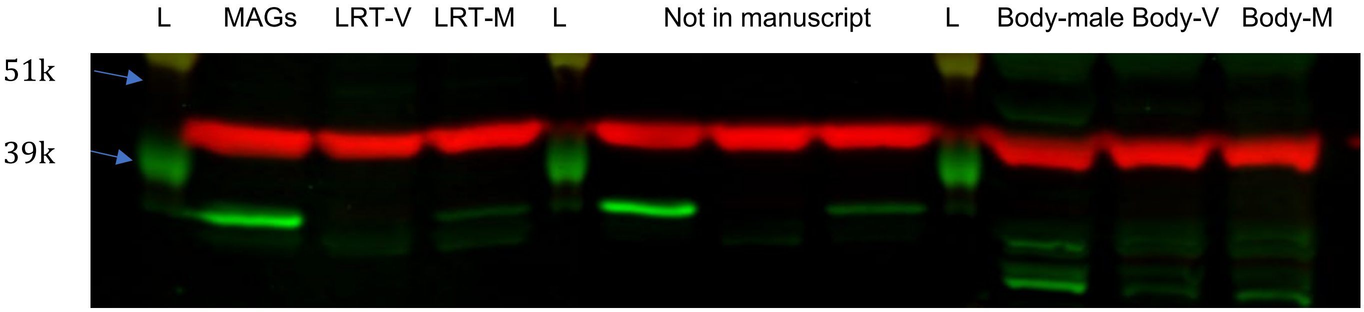

L = Ladder (SeeBlue™ Plus2 Pre-stained Protein Standard)

Red channel in gray scale (α-Actin)

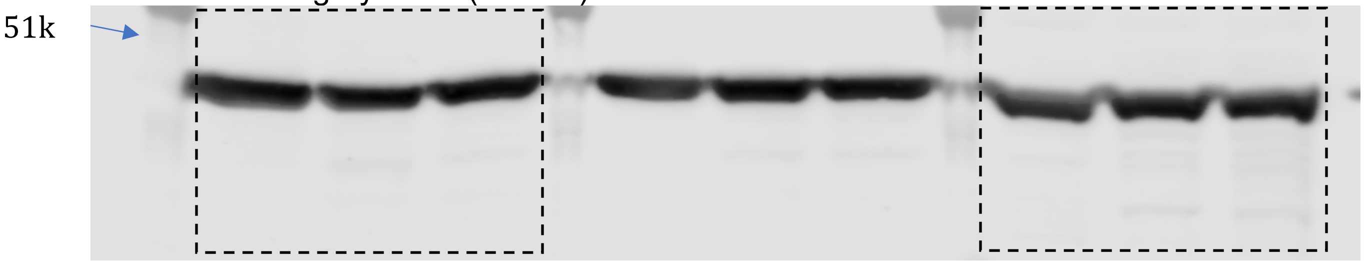

Green channel (α-EPP)

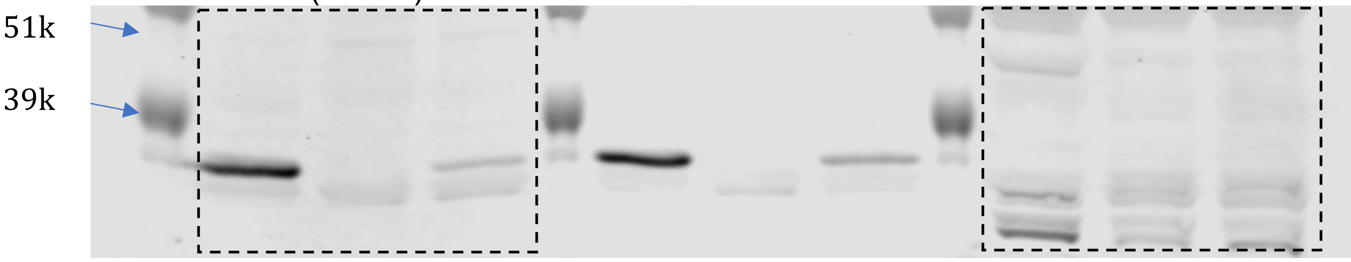

Supplementary Figure 1: Uncropped images used in generating Fig. 2d. Dashed boxes show how the images were cropped to generate Fig. 2d.
