# Supplementary material for: A male steroid controls female sexual behaviour in the malaria mosquito
Source: Nature. 2022 Jul 6;608(7921):93–7. doi: 10.1038/s41586-022-04908-6 (PMC9352575; doi:10.1038/s41586-022-04908-6)
Supplement: Supplementary file 2 — Reporting Summary [file 41586_2022_4908_MOESM2_ESM.pdf]

nature portfolio

Corresponding author(s): Flaminia Catteruccia

Last updated by author(s): Apr 26, 2022

Reporting Summary

Nature Portfolio wishes to improve the reproducibility of the work that we publish. This form provides structure for consistency and transparency in reporting. For further information on Nature Portfolio policies, see our Editorial Policies and the Editorial Policy Checklist.

Statistics

For all statistical analyses, confirm that the following items are present in the figure legend, table legend, main text, or Methods section.

n/a	Confirmed	
☐	☒	The exact sample size (*n*) for each experimental group/condition, given as a discrete number and unit of measurement
☐	☒	A statement on whether measurements were taken from distinct samples or whether the same sample was measured repeatedly
☐	☒	The statistical test(s) used AND whether they are one- or two-sided<br>*Only common tests should be described solely by name; describe more complex techniques in the Methods section.*
☒	☐	A description of all covariates tested
☐	☒	A description of any assumptions or corrections, such as tests of normality and adjustment for multiple comparisons
☐	☒	A full description of the statistical parameters including central tendency (e.g. means) or other basic estimates (e.g. regression coefficient) AND variation (e.g. standard deviation) or associated estimates of uncertainty (e.g. confidence intervals)
☐	☒	For null hypothesis testing, the test statistic (e.g. *F*, *t*, *r*) with confidence intervals, effect sizes, degrees of freedom and *P* value noted<br>*Give P values as exact values whenever suitable.*
☒	☐	For Bayesian analysis, information on the choice of priors and Markov chain Monte Carlo settings
☒	☐	For hierarchical and complex designs, identification of the appropriate level for tests and full reporting of outcomes
☒	☐	Estimates of effect sizes (e.g. Cohen's *d*, Pearson's *r*), indicating how they were calculated

Our web collection on statistics for biologists contains articles on many of the points above.

Software and code

Policy information about availability of computer code

Data collection	Xcalibur 4.4, Design and Analysis 2.4.3, Image Studio 5.2
Data analysis	Xcalibur 4.4, Tracefinder 4.1, Proteowizard 3.0.20287, Comet 3.2, Percolator 3.05, Limelight 2.2.0, Image Studio 5.2, Design and Analysis 2.4.3, Graphpad 9.0., HISAT2 2.0.5, Samtools 1.3.1, htseq-count 0.9.1, DESeq2 1.28.1, R 4.0.3, PSI-Blast 2.8.1, Primer-BLAST (https://www.ncbi.nlm.nih.gov/tools/primer-blast/)

For manuscripts utilizing custom algorithms or software that are central to the research but not yet described in published literature, software must be made available to editors and reviewers. We strongly encourage code deposition in a community repository (e.g. GitHub). See the Nature Portfolio guidelines for submitting code & software for further information.

Data

Policy information about availability of data

All manuscripts must include a data availability statement. This statement should provide the following information, where applicable:

- Accession codes, unique identifiers, or web links for publicly available datasets
- A description of any restrictions on data availability
- For clinical datasets or third party data, please ensure that the statement adheres to our policy

All original western blots are provided in the Supplementary Information.
The MS proteome data were deposited to the ProteomeXchange Consortium (http://proteomecentral.proteomexchange.org) via the PRIDE partner repository (https://www.ebi.ac.uk/pride/) with the dataset identifier PXD032157.
The RNAseq data were deposited to the Gene Expression Omnibus repository (https://www.ncbi.nlm.nih.gov/geo/) under the series record GSE198665.
Other datasets generated during and/or analysed during the current study are available from the corresponding author on reasonable request.

Field-specific reporting

Please select the one below that is the best fit for your research. If you are not sure, read the appropriate sections before making your selection.

☒ Life sciences ☐ Behavioural & social sciences ☐ Ecological, evolutionary & environmental sciences

For a reference copy of the document with all sections, see nature.com/documents/nr-reporting-summary-flat.pdf

Life sciences study design

All studies must disclose on these points even when the disclosure is negative.

Sample size	We did not perform power analysis for sample size calculation. Sample size was shaped by a combination of time, feasibility, and prior experience to obtain maximum statistical power with reasonable resource. The following factors had a large impact on sample size: (1) the maximum number of mosquitoes housed in a laboratory cage (small cage n=100, medium cage n=200), (2) blood-feeding / mating rates, injection survival rates, and (3) the maximum number of mosquito can be processed/dissected for each time point.
Data exclusions	No data were excluded from the analysis
Replication	The experimental findings were confirmed with three or more replicates with the exception of western blot (2 biological replicates).
Randomization	Mosquitoes were randomly allocated into experimental groups by aspiration
Blinding	Investigators were blinded to group allocation during data analysis. The investigators were not blinded to group allocation during mosquito sample processing, and the mosquito cages were labeled with group allocation information that can be seen by the investigators.

Reporting for specific materials, systems and methods

We require information from authors about some types of materials, experimental systems and methods used in many studies. Here, indicate whether each material, system or method listed is relevant to your study. If you are not sure if a list item applies to your research, read the appropriate section before selecting a response.

Materials & experimental systems

n/a	Involved in the study	
☐	☒	Antibodies
☒	☐	Eukaryotic cell lines
☒	☐	Palaeontology and archaeology
☐	☒	Animals and other organisms
☒	☐	Human research participants
☒	☐	Clinical data
☒	☐	Dual use research of concern

Methods

n/a	Involved in the study	
☒	☐	ChIP-seq
☒	☐	Flow cytometry
☒	☐	MRI-based neuroimaging

Antibodies

Antibodies used	Primary Antibody:<br>(1) Anti-EPP, custom ordered from Genscript NJ, US (see methods for details)<br>(2) Anti-Actin antibody [MAC 237] (ab50591) Abcam<br><br>Secondary Antibody:<br>(1) IRDye® 800CW Donkey anti-Rabbit IgG Secondary Antibody, P/N: 926-32213<br>(2) IRDye® 680LT Goat anti-Rat IgG Secondary Antibody, P/N: 926-68029
Validation	Anti-EPP was validated by western blot using recombinant EPP and male accessory glands samples. Cross-reactivity was verified by western using virgin female mosquito samples.<br>Anti-Actin antibody was validated for use in Anopheles gambiae mosquitoes by Werling et al., 2019 Cell 177, 315-325.<br>Secondary antibodies were confirmed by Werling et al., 2019 Cell 177, 315-325.

Animals and other organisms

Policy information about studies involving animals; ARRIVE guidelines recommended for reporting animal research

Laboratory animals	Anopheles gambiae G3 strain, male and female, age: 1 day old and 4 days old.
Wild animals	No wild animals were used in this study

Field-collected samples	No field-collected samples were used in this study
Ethics oversight	No ethical approval or guidance was required

Note that full information on the approval of the study protocol must also be provided in the manuscript.
