# Supplementary material for: A male steroid controls female sexual behaviour in the malaria mosquito
Source: Nature. 2022 Jul 6;608(7921):93–7. doi: 10.1038/s41586-022-04908-6 (PMC9352575; doi:10.1038/s41586-022-04908-6)
Supplement: Supplementary file 6 — Supplementary Table 3 P values in Extended Data Fig. 3. [file 41586_2022_4908_MOESM6_ESM.pdf]

Supplementary Table 3: *P* values in Extended Data Fig. 3.

	MISO	*HPX15*	*AGAP006421*	*AGAP004263*	*AGAP007041*	*EcR-A*	*EcR-B*	*E75*	*HR3*	*HR4*	*Vg*
**20E vs 3D20E**	0.026	0.05	0.01	0.0034	0.5572	0.0166	0.0786	0.2628	0.0002	4.19E-05	0.0018
**20E vs 10% EtOH**	0.0478	0.0833	0.0654	0.0444	0.001	0.4705	0.8906	0.0973	0.0002	6.35E-07	0.0011
**3D20E vs 10% EtOH**	0.0008	0.0034	0.0154	0.0016	0.0141	0.0027	0.0382	0.1933	0.0019	0.0974	0.0847
