# Supplementary material for: A male steroid controls female sexual behaviour in the malaria mosquito
Source: Nature. 2022 Jul 6;608(7921):93–7. doi: 10.1038/s41586-022-04908-6 (PMC9352575; doi:10.1038/s41586-022-04908-6)
Supplement: Supplementary file 7 — Sequences of dsRNA constructs (target gene-specific sequences). [file 41586_2022_4908_MOESM7_ESM.docx]

**Supplementary Data 1: Sequences of dsRNA constructs (target gene-specific sequences).**

ds*GFP*: ACGTAAACGGCCACAAGTTCAGCGTGTCCGGCGAGGGCGAGGGCGATGCCACCTACGGCAAGCTGACCCTGAAGTTCATCTGCACCACCGGCAAGCTGCCCGTGCCCTGGCCCACCCTCGTGACCACCCTGACCTACGGCGTGCAGTGCTTCAGCCGCTACCCCGACCACATGAAGCAGCACGACTTCTTCAAGTCCGCCATGCCCGAAGGCTACGTCCAGGAGCGCACCATCTTCTTCAAGGACGACGGCAACTACAAGACCCGCGCCGAGGTGAAGTTCGAGGGCGACACCCTGGTGAACCGCATCGAGCTGAAGGGCATCGACTTCAAGGAGGACGGCAACATCCTGGGGCACAAGCTGGAGTACAACTACAACAGCCACAACGTCTATATCATGGCCGACAAGCAGAAGAACGGCATCAAGGTGAACTTCAAGATCCGCCACAACATCGAGGACGGCAGCGTGCAGCTCGCCGACCACTACCAGCAGAACAA

ds*EPP*:

GCAACAGAACGGTGGCAAAATGTTCGGTGGCAATAAAACGATGGACGACATTCCGCTGGAAAAGTTGCACGAGCTGGTGAAAAAGCGTACCAACATGCAGGTGATCCCGGGAGATCAGTGCGCCTCACAGTCGCAGTCCGTACCGCGGACGGAATCGGGCACGCGCAAGGTGTACGTCATGCGACACGGCGAGCGGATCGATTTTACCTTTGGTTCGTGGGTACCGTACTGCTTCGATGAGGCTGGCAACTACGTCCGCAAAGATCTGAACATGCCCACATCGTTGCCCACACGTAAACCCAGCCTCTGGCAGAAGGATTCACCACTGACGAACGTGGGTCACTATCAGGCACGGCTCGTCC

ds*EcK1*:

TTTCTCTTTCAAACGACACGGGCAACGGAGAGCTACCAACAATTAGTCGAACAAGGAATGCCTCAACTAACCAAGTTTCTTGAGCACAAACCTGGGTTTGAAAATGAACTAAAAGCGCTGAGCAAAATTCGTCCAAAAACGAAATTTCTGATTGAAACGCTTCTGCAGCCAATCGAGCCGATGGGACTGATTACACATACGGACTTTTGGTGCAACAATTTGCTGTTCCGCAGTGAACCGGACGAAGAACGTACCGACAGTTGCACTATTCTAGACTGGCAAATGGTAACATATAGCCGTCCAACGAACGATCTTGCCTTGCTGCTGATTTCGTCCATTCCGTCCAACACTCGACGTCAGCACAC

ds*EcK2*:

TAGCGGTGAGTGATTGAGCAGCAGCAGCAGCAATAACCGCCAAAATACTACACACTACAGTGTGTGTGTGTGCGTGTGAGTTAATTTTGCGTAATTTTGATGCGTGTGCGTGTACCTGTGAGTGAGTGAAGTGTGTGGTAAGCGAAATAGGCAAGTGTGGCTTTATAAACATACACAAACACACCCACCCGCGAAAGACCAGCAACAGCAAGAGCAAAAGCGATTCCGGTTGGAGCGAACTTGCCCCAAAACAAACAGAAAACAAACGAACAAAAAACCCGCTCTCCCCCCATCGTCGCCGCCTTCACATGCAAGCCCGCACAGCCGTCAGGATGAAGCAAGAAGTGGCGGTCGGCATGTACGTGGACGAGGATCGGGACGTGTACATGATCGAGCGGAAGGACACGATCGTCGACATCATCCGGATGCAGCGGAAGGCGGGCCTGTGGCAGACGCTGTCGGTCGACCTGGTCGACTGCGGGCCGGGCAGCCGGGAGGGCGACAACTACATGTCGATCATCAAGCGGGTAAAGGCCCACTGTAAGGCGAAATCGGT
